# Multimodal Integration of Genomic Data Reveals Regulatory Mechanisms at the Polycystic Ovary Syndrome (PCOS)-Associated 12q13.2 Locus

**DOI:** 10.3390/ijms262211184

**Published:** 2025-11-19

**Authors:** R. Alan Harris, Jan M. McAllister, Jerome F. Strauss

**Affiliations:** 1 Human Genome Sequencing Center, Baylor College of Medicine, Houston, TX 77030, USA; 2 Department of Molecular and Human Genetics, Baylor College of Medicine, Houston, TX 77030, USA; 3 Departments of Pathology and Ob/Gyn, Penn State Hershey College of Medicine, Hershey, PA 17033, USA; jxm63@psu.edu; 4 Department of Obstetrics and Gynecology, Perelman School of Medicine, University of Pennsylvania, Philadelphia, PA 19104, USA

**Keywords:** polycystic ovary syndrome, human ovarian theca cells, androgens, multimodal, whole exome sequencing, single-cell RNA sequencing, STARR-seq, eQTL

## Abstract

Polycystic ovary syndrome (PCOS) is a complex endocrine disorder affecting reproductive-aged women. Previous studies have identified genomic associations at chromosome 12q13.2, but the functional mechanisms underlying these associations remain unclear. We integrated three complementary datasets: (1) WES-identified single nucleotide variants (SNVs) from PCOS and normal theca cells with association testing for forskolin-stimulated androgen production, (2) STARR-seq enhancer activity data with eQTL colocalization analysis, and (3) scRNA-seq expression data comparing forskolin-stimulated PCOS and normal theca cells. We previously identified haplotypes involving 10 SNVs at 12q13.2 containing *RPS26*/*RAB5B*/*SUOX* that are significantly associated with forskolin-stimulated androgen production. The identified haplotypes were further shown to associate with PCOS in a whole genome sequencing (WGS) cohort. Other studies have recently found the enhancer variant rs1081975 demonstrated perfect colocalization (PP = 1.0) with *RPS26*/*RAB5B*/*SUOX* eQTLs. Our scRNA-seq analysis revealed differential expression patterns for key genes. *RAB5B* showed a forskolin response upregulation in normal cells but an impaired response in PCOS. *SUOX* exhibited opposite forskolin responses between normal and PCOS cells. *PA2G4*, an androgen corepressor in the locus, was upregulated in normal untreated cells. *ERBB3*, an epidermal growth factor receptor in the locus, was downregulated in normal forskolin treated cells. The integration of multimodal genomic data provides functional validation of PCOS-associated variants at 12q13.2, revealing coordinated dysregulation of vesicular trafficking (*RAB5B*), androgen receptor regulation (*PA2G4*), and metabolic processes (*SUOX*) in PCOS theca cells.

## 1. Introduction

Polycystic Ovary Syndrome (PCOS) affects 5–15% of reproductive-aged women [1]. It is characterized by bilaterally enlarged ovaries containing multiple cystic follicles, and dysfunction of thecal cells resulting in hyperandrogenemia, anovulation, and infertility. PCOS is a complex genetic disorder with substantial heritability, as evidenced by familial clustering and twin studies [1,2,3]. Genome-wide association studies (GWAS) have identified multiple susceptibility loci for PCOS, including regions involved in gonadotropin secretion, steroidogenesis, and metabolic pathways [4,5,6]. The chromosome 12q13.2 region has emerged as a locus of particular interest, containing several genes implicated in PCOS pathogenesis including *ERBB3*, *PA2G4*, *RPS26*, *RAB5B*, and *SUOX*. However, the functional mechanisms by which genetic variants at this locus contribute to PCOS pathophysiology remain incompletely understood.

Ovarian theca cells play a central role in PCOS pathogenesis through their production of excess androgens [7,8,9,10]. In PCOS these cells exhibit intrinsic abnormalities in steroidogenic enzyme expression and activity leading to enhanced androgen synthesis [10]. Understanding the genetic basis of these cellular abnormalities is critical for elucidating PCOS pathogenesis.

Based on our review of the recent publication by Sankaranarayanan and colleagues (2025) [11] and building on their data, we integrated advanced genomic technologies that enable multimodal approaches to characterize disease mechanisms. (1) Expression quantitative trait locus (eQTL) mapping identifies genetic variants that influence gene expression levels [11]. (2) Self-transcribing active regulatory region sequencing (STARR-seq) provides functional validation of enhancer elements [11]. (3) Single-cell RNA sequencing (scRNA-seq) reveals cell-type-specific expression patterns and responses to stimulation [10]. The integration of these complementary approaches offers the potential to bridge the gap between genetic association and functional mechanisms, addressing the multifactorial complexity of PCOS genetics emphasized in a recent review [12].

In this study, we present an integrated analysis of the 12q13.2 PCOS-associated locus using three complementary datasets. Our approach combines previously published whole exome sequencing (WES) data identifying single nucleotide variants (SNVs) associated with androgen production in theca cells [6], STARR-seq enhancer activity data with eQTL colocalization analysis [11], and scRNA-seq sequencing comparing gene expression patterns between PCOS and normal theca cells [10].

Unlike previous studies analyzing individual datasets, this is the first comprehensive integration directly linking 12q13.2 WES variants to STARR-seq enhancer activity and functional scRNA-seq validation of target genes. This analysis reveals coordinated dysregulation of three distinct pathways, a mechanistic insight unavailable from isolated dataset analysis.

## 2. Results

**Figure 1:**
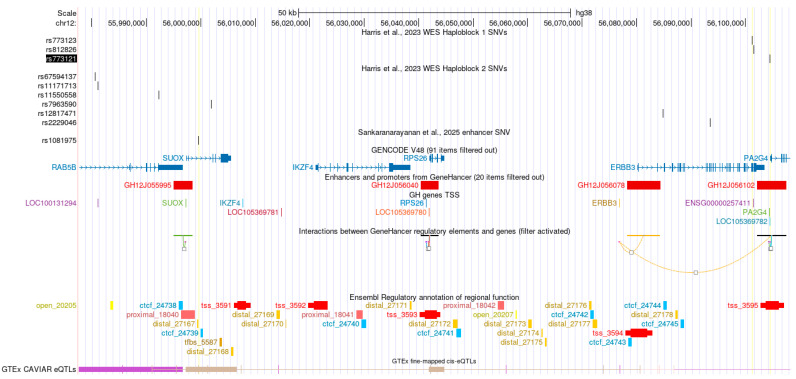
UCSC Genome Browser view of the region of interest examined including data from [6,10,11] together with relevant browser tracks.

The genomic data integrated to perform this multimodal analysis are shown in Figure 1. It is also viewable as an interactive session in the UCSC Browser (https://genome.ucsc.edu/s/Rharris1/hg38.PCOS.multimodal, accessed on 14 November 2025) [13].

### 2.1. Genetic Associations at 12q13.2

WES identified 10 SNVs in 12q13.2 significantly associated with forskolin-stimulated androgen production in theca cell cultures [6] (Table S1). These variants clustered within a 150 kb region at chromosome 12q13.2, encompassing genes previously implicated in PCOS. Furthermore, we determined that 7 of these SNVs were in one haploblock with the remaining 3 being in a separate haploblock.

We analyzed the 10 SNVs on chromosome 12 that were significantly associated with thecal cell androgen production in whole genome sequencing (WGS) data from a family based PCOS cohort [14]. Based on the HBAT [15] test, the haplotype consisting of the minor alleles for rs773123, rs812826, and rs773121, and the major alleles for the remaining SNVs were found preferentially in women with PCOS and elevated androgen levels (*p* = 0.0583).

Two variants in the haplotype associated with PCOS were predicted to have functional consequences based on genomic context: a *ERBB3* missense variant (rs773123 https://www.ncbi.nlm.nih.gov/snp/rs773123, accessed on 14 November 2025) and a *PA2G4* promoter variant (rs773121 https://www.ncbi.nlm.nih.gov/snp/rs773121, accessed on 14 November 2025) [6]. The *PA2G4* promoter region corresponds to the GeneHancer [16] regulatory element GH12J056102 (https://www.genecards.org/cgi-bin/carddisp.pl?gene=PA2G4&keywords=GH12J056102#genomic_location, accessed on 14 November 2025), which has predicted enhancer functions for both *ERBB3* and *RAB5B* [6].

We annotated the SNVs with CADD v1.6 [17] (Table S2). A CADD PHRED score of 10 predicts a SNV is among the 10% most functionally significant changes in the human genome while a CADD PHRED score of 20 indicates the change is among the 1% most functional changes. The *ERBB3* missense variant (rs773123 https://www.ncbi.nlm.nih.gov/snp/rs773123, accessed on 14 November 2025) had a CADD PHRED score of 24.1. The *PA2G4* promoter variant (rs773121 https://www.ncbi.nlm.nih.gov/snp/rs773121, accessed on 14 November 2025) had a CADD PHRED score of 14.01.

### 2.2. Enhancer Activity and eQTL Colocalization

STARR-seq analysis identified an active regulatory element within the 12q13.2 region [11]. The enhancer variant rs1081975 (https://www.ncbi.nlm.nih.gov/snp/rs1081975, accessed on 14 November 2025) demonstrated perfect colocalization (posterior probability = 1.0) with eQTLs affecting *RPS26*, *RAB5B*, and *SUOX* expression [11]. This variant was located 3 bp from the Ensembl Regulatory [18] distal_27167 predicted enhancer. It also coincided with three GTEx [19] eQTLs affecting *RAB5B* in heart atrial appendage, *RPS26* in uterus, and *RPS26* in minor salivary gland as shown in the UCSC interactive session.

### 2.3. Single-Cell Expression Patterns

scRNA-seq revealed distinct differential expression patterns for genes within the 12q13.2 region when comparing PCOS and normal theca cells either untreated or forskolin treated (Table 1 and Table S3) [10]. *RAB5B* showed lower baseline expression in normal untreated theca cells and upregulation in normal forskolin treated theca cells with no significant forskolin response detected in PCOS cells. We have previously shown RAB5B accumulates in the nuclei of PCOS theca cells treated with forskolin [20], but this is the first evidence that forskolin treatment also upregulates *RAB5B*. *SUOX* showed opposite patterns in forskolin treated normal and PCOS theca cells with upregulation in normal cells and downregulation in PCOS cells. *PA2G4* showed upregulation in untreated normal theca cells and no significant differential expression in other conditions. *ERBB3* showed downregulation in forskolin treated normal theca cells and no significant differential expression in other conditions. These patterns indicate coordinated dysregulation of cellular responses to cAMP stimulation in PCOS theca cells compared to untreated cells [10].

Although individual log2 fold changes are modest, coordinated changes across a network can still drive biological function, especially when supported by genetic association and orthogonal validation such as eQTL colocalization. Recent work demonstrates that integration of GWAS with scRNA-seq data reveals disease-relevant cell types and pathways [21]. Furthermore, scRNA-seq data analysis reveals functionally relevant biomarkers and their regulatory footprints through pathway and protein–protein interaction analyses [22]. The directional consistency of *RAB5B*, *PA2G4*, and *SUOX* expression changes across PCOS versus normal theca cells is corroborated by independent validation from genetic association at this locus and eQTL colocalization, providing orthogonal evidence supporting the biological relevance of these subtle transcriptional changes.

### 2.4. STRING Analysis

**Figure 2:**
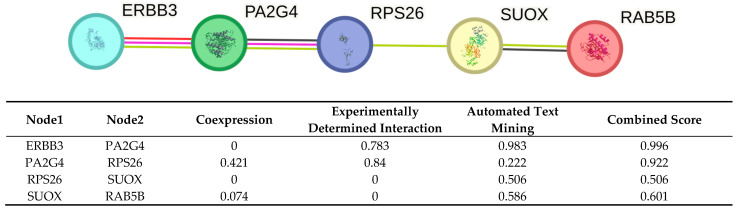
STRING analysis of the genes identified as differentially expressed in our scRNA-seq data together with *RPS26* that was identified by eQTL analysis. Column descriptions: Coexpression: score based on transcriptional coexpression across multiple experimental conditions or tissues; reflects coordinated gene regulation. Experimentally determined interaction: score derived from curated experimental data (e.g., yeast two-hybrid, co-immunoprecipitation) indicating direct physical binding. Automated text mining: score based on statistical co-mentions in scientific articles, extracted via natural-language processing of abstracts and full-text. Combined score: composite confidence score integrating all above channels into a single metric. All scores are scaled between 0 and 1.

We used the STRING: functional protein association networks database [23] to examine relationships among the genes identified as differentially expressed in our scRNA-seq data together with *RPS26* that was identified by eQTL analysis [11] (Figure 2).

1. ERBB3–PA2G4 (0.996): Despite no coexpression evidence, the very high experimental and text mining scores yield a near maximal combined score. This suggests a well validated association between ERBB3 and PA2G4, heavily supported by curated experiments and literature.
2. PA2G4–RPS26 (0.922): Moderate coexpression and strong experimental support results in a high combined score. Text mining contributes less, indicating fewer co-mentions.
3. RPS26–SUOX (0.506): With no coexpression or experimental support, this association relies solely on text mining at a borderline combined score. This suggests a putative link from literature.
4. SUOX–RAB5B (0.601): This pair has low coexpression and no direct experimental evidence. The interaction is inferred primarily through literature co-mentions (0.586), yielding a moderate combined score.

### 2.5. Integrated Mechanistic Model

**Figure 3:**
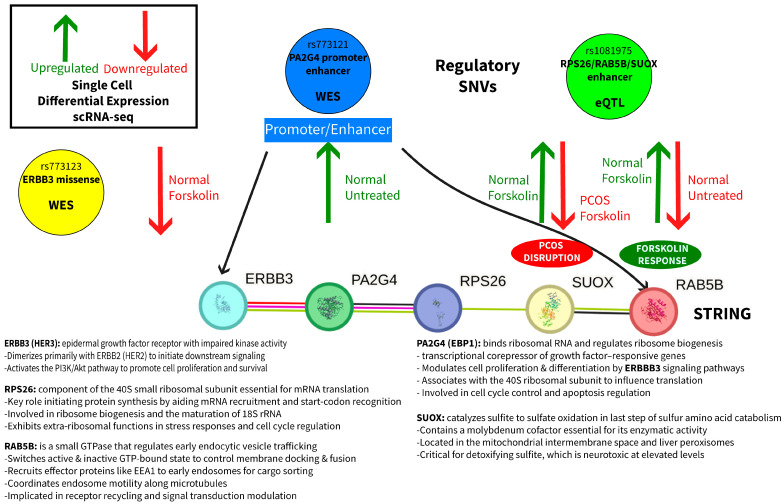
Integrated Mechanistic Model from genetic, enhancer, and expression data. Green upward arrows indicate upregulation, and red downward arrows indicate downregulation based on scRNA-seq data. Text labels within circles indicate the modality that detected each variant (WES or eQTL). Differential responses to forskolin treatment reveal disrupted cellular signaling in PCOS theca cells compared to normal controls.

The convergent evidence from genetic, enhancer, and expression data supports a model of coordinated dysfunction at the 12q13.2 locus (Figure 3):

1. Genetic: SNVs in a promoter/enhancer (rs773121) and enhancer (rs1081975) affect baseline gene expression and forskolin treatment responsiveness.
2. Regulatory disruption: Promoter/enhancer SNVs alter the normal coordination between RAB5B vesicular trafficking, PA2G4 androgen receptor regulation, and SUOX metabolic functions.
3. Cellular dysfunction: Loss of coordinated responses to hormonal stimulation results in impaired androgen regulation and cellular homeostasis in PCOS theca cells.

## 3. Discussion

This multimodal integration provides functional validation of genetic associations at the PCOS-associated 12q13.2 locus. The convergent evidence from three independent datasets, genetic association, enhancer activity, and scRNA-seq, supports a model where regulatory variants coordinately disrupt multiple cellular pathways in PCOS theca cells, leading to hyperandrogenemia, a cardinal feature of PCOS.

*RAB5B* showed lower baseline expression in normal untreated theca cells and upregulation in normal forskolin treated theca cells suggesting this is part of the normal theca cell response to forskolin treatment, which mimics the action of LH by stimulating cAMP production, leading to increased thecal androgen synthesis. We have previously shown RAB5B accumulates in the nuclei of PCOS theca cells treated with forskolin [20], but this is the first evidence that forskolin treatment also upregulates *RAB5B*. The greater resolution of single cell compared to bulk RNA approaches may account for why *RAB5B* expression changes in response to forskolin treatment have not been previously detected.

The perfect eQTL colocalization (PP = 1.0) of rs1081975 with *RPS26/RAB5B/SUOX* expression in the context of PCOS provides strong evidence for functional regulatory effects [11]. This is complemented by our single-cell data demonstrating that *SUOX* shows opposite responses to forskolin stimulation between PCOS and normal cells [10], validating the predicted functional consequences of the genetic variant.

The proposed mechanism involves dysregulation of vesicular trafficking (*RAB5B*), androgen receptor coexpression (*PA2G4*), and metabolic processes (*SUOX*). This is consistent with the known role of *PA2G4* as a transcriptional corepressor of androgen receptor regulated genes and the involvement of *RAB5B* in endocytic trafficking pathways critical for cellular signaling.

The STRING analysis supports our proposed network. ERBB3 and PA2G4 interact with very high confidence (0.996), backed by strong experimental evidence. PA2G4 and RPS26 show robust interaction (combined score 0.922) through moderate coexpression (0.421) and strong experimental support (0.840). This suggests PA2G4 repression of androgen-regulated genes is linked to ribosomal function through RPS26, potentially explaining the altered protein synthesis and stress responses seen in PCOS theca cells. The SUOX-RAB5B link is weaker (0.601), relying mainly on literature mentions and needs direct experimental confirmation. This coordinated regulation may underlie the altered protein synthesis and stress response phenotypes documented in PCOS theca cells [10].

Our previous research revealed that DENND1A.V2, a truncated splice variant of the *DENND1A* gene located within a chromosome 9 GWAS identified PCOS-associated locus, is upregulated in PCOS theca cells [9]. Moreover, forced expression of the DENND1A.V2 protein in normal theca cells increased their androgen biosynthesis by driving expression of key steroidogenic proteins involved in androgen synthesis, including CYP11A1 and CYP17A1, by stimulating promoter activity. DENND1A.V2 is produced by exonization of sequences in intron 20 of the *DENND1A* gene, which generates a unique exon 20A, that encodes the 33 AA C-terminus, which is hydrophobic, with a basic pI. Mass spectrometry studies deposited in public databases show that the C-terminus of DENND1A.V2 can undergo post-translational modification by phosphorylation (PhosphoSitePlus V6.5.9). The DENND1A.V2 splice variant has not yet been detected in laboratory animals or domestic species. However, the *Dennd1a* gene has essential functions in mice including ovarian morphogenesis since *Dennd1a* null embryos die around embryonic day 14.5 with abnormal ovarian morphology secondary to impaired fetal germ cell migration and differentiation [24].

DENND1A.V2 is associated with plasma membrane domains where gonadotropin receptors and RAB5B are localized and it is internalized in endocytic vesicles that traffic to the nucleus of theca cells upon forskolin stimulation [20]. This suggests that DENND1A.V2 is part of a novel signal transduction network including genes on 12q13 engaged in vesicular trafficking [20] of cell surface receptors for gonadotropins and enzymes producing intracellular second messengers, which may account for the functional and structural ovarian phenotypes characteristic of PCOS (e.g., thecal cell hypertrophy, hyperandrogenemia, follicular growth arrest, and anovulation) as well as alterations in cellular metabolism (e.g., obesity) frequently associated with PCOS [20].

The integration of genetic, enhancer activity, and single cell data provides convergent evidence for functional mechanisms at the PCOS-associated 12q13.2 locus. The coordinated dysregulation of *RAB5B*, *PA2G4*, and *SUOX* in PCOS theca cells supports a model where regulatory variants disrupt multiple cellular pathways involved in vesicular trafficking, androgen production, and metabolic function. This multimodal approach demonstrates the value of integrating complementary genomic datasets to bridge the gap between genetic association and functional mechanisms in complex disease.

Although biological replicates were limited, they were sufficient for statistical rigor. The initial WES analyses were performed on 16 theca preparations consisting of 9 PCOS and 7 normal samples. However, the association was strengthened by independent replication of WES-identified haplotypes in a separate family cohort of 77 families. scRNA-seq was performed on 5 PCOS and 5 normal theca cell preparations. However, stringent quality control in scRNA-seq (>5% mitochondrial filtering, STACAS batch correction at confidence 0.99), and high statistical power from analyzing a total of 15,565 individual cells with a minimum of 2654 cells for any comparison enabled Bonferroni correction and robust significance thresholds.

The PA2G4–RAB5B–SUOX network is supported by the STRING database, which aggregates multiple published experiments demonstrating these interactions. Functional validation by reporter assays or co-immunoprecipitation would strengthen mechanistic conclusions. However, direct protein–protein interactions in theca cells specifically remain to be experimentally validated.

## 4. Materials and Methods

Three complementary datasets were integrated for this analysis:

1. WES-identified SNVs from PCOS and normal theca cells with association testing for forskolin-stimulated androgen production (Harris, et al. 2023 [6])
2. STARR-seq enhancer activity data with eQTL colocalization analysis (Sankaranarayanan, et al. 2025 [11])
3. scRNA-seq expression data comparing forskolin-stimulated PCOS and normal theca cells (Harris, et al. 2023 [10])

Summarized methods relevant to the current paper are shown below. See the methods section of the cited papers for further details.

Harris, et al. 2023 [6]—Loci on chromosome 12q13.2 encompassing ERBB3, PA2G4, and RAB5B are associated with polycystic ovary syndrome.

### 4.1. Theca Cell Preparations and Culture

Human theca tissue was obtained from luteal-phase follicles of age-matched (38–41 years) women undergoing hysterectomy after informed consent and IRB approval at Virginia Commonwealth University and Penn State College of Medicine. The diagnosis of PCOS adhered to NIH consensus criteria: hyperandrogenemia or hyperandrogenism, oligo-ovulation, and exclusion of other androgen-excess etiologies. All PCOS ovaries exhibited multiple subcortical follicles <10 mm. Normal tissues came from regularly cycling, fertile women with 21–35-day cycles and no hyperandrogenism. Primary cells were enzymatically isolated, expanded to second passage, cryopreserved, and then cultured under identical media and split-ratio conditions to fourth passage (31–38 doublings). Nine PCOS and seven normal preparations from unrelated, individuals of European ancestry were analyzed. Preparations were characterized by basal and forskolin (Calbiochem, San Diego CA, USA) (20 μM, 16 h)-stimulated DHEA production measured by ELISA (DRG International, Springfield, NJ, USA) and normalized to 10^6^ cells.

### 4.2. Whole Exome Sequence Analysis of Normal and PCOS Theca Cell DNA

Genomic DNA from flash-frozen fourth-passage theca cells was extracted using a Qiagen kit and subjected to 100× coverage WES (Agilent SureSelect 51M, Illumina HiSeq 2000). Reads were aligned to GRCh37/hg19 with BWA-MEM [25] v0.7.12, followed by duplicate marking (Picard), indel realignment, left-alignment, and base quality recalibration with GATK [26] walkers.

### 4.3. Linkage Disequilibrium

LDlink SNPclip (https://ldlink.nih.gov/?tab=snpclip, accessed on 14 November 2025) was used to assess r^2^ ≥ 0.5 among nine DHEA-associated SNVs in Europeans from 1000 Genomes, identifying two LD groups for haplotype inference via LDlink LDhap (https://ldlink.nih.gov/?tab=ldhap, accessed on 14 November 2025).

### 4.4. Statistical Analysis

Age and DHEA production (basal and forskolin-stimulated) were compared between PCOS and controls by a Wilcoxon rank-sum test. WES variants were filtered to remove non-variable sites across samples, yielding 441 variants; those with ≥2 minor-allele carriers were tested (*n* = 252) for association with forskolin-stimulated DHEA by the Wilcoxon test (*p* < 0.05 threshold; no Bonferroni correction).

### 4.5. SNV Analyses of a PCOS Cohort

Ten chromosome-12 SNVs associated with theca cell androgen production were analyzed in 318 individuals of European ancestry from 77 families (90 PCOS, 5 hyperandrogenemic, 76 unaffected) by transmission disequilibrium test in PLINK v1.90 [27] and haplotype association by FBAT [15] HBAT.

### 4.6. Functional Annotations

Potential functional consequences of the SNVs were examined using Combined Annotation-Dependent Depletion (CADD) v1.6 [17] and GeneHancer [16].

Sankaranarayanan, et al. 2025 [11]—Gene regulatory activity associated with polycystic ovary syndrome revealed DENND1A-dependent testosterone production.

### 4.7. Selection of GWAS Regions for Targeted STARR-Seq Assays

SNVs from PCOS risk loci identified by GWAS publications prior to 2019, 27 total, were filtered for genome-wide significance (*p*  <  5  ×  10^−8^), yielding 19 loci. Each identified SNV and all SNVs in linkage disequilibrium (r^2^  >  0.8) were retrieved, and overlapping bacterial artificial chromosomes (BACs) or fosmids were selected. Three loci (ZBTB16, MAPRE, and ERBB3) were excluded due to recombination or clone unavailability. The remaining 16 loci were represented by 18 BACs and 2 fosmids sourced from BACPAC Genomics (Children’s Hospital & Research Center Oakland). Clones were propagated in *E. coli*, purified (NucleoBond Xtra BAC (Machery-Nagel); FosmidMAX (Lucigen)), and integrity was confirmed by Illumina MiSeq sequencing of NEBNext Ultra II FS–prepared libraries barcoded per clone. Two regions failed validation and were omitted, leaving 14 loci spanning ~3 Mb.

### 4.8. STARR-Seq Reporter Plasmid Construction

Each validated BAC or fosmid was sheared to ~400 bp on a Covaris S220, end-repaired, A-tailed, and ligated to custom adapters (NEBNext DNA Library Prep). Fragments were PCR-amplified with KAPA HiFi HotStart using TS2SS-F/R primers to introduce Gibson assembly overlaps. The STARR-seq ORI vector (Addgene #99296) was linearized with AgeI/SalI, size-confirmed (~3.6 kb), and purified. Adapted inserts were assembled into the vector using NEBuilder HiFi, ethanol-precipitated, and transformed into *E. cloni* 10G SUPREME cells by electroporation. Colonies were expanded in 1 L LB  +  carbenicillin, and plasmid libraries were isolated (NucleoBond PC 10000 EF). Individual libraries were pooled equimolarly, quality-checked on an Agilent TapeStation, and quantified by Qubit.

### 4.9. STARR-Seq Assay Library Sequencing

Pooled STARR-seq plasmids (20 ng) were PCR-amplified with KAPA HiFi (208-F Index7 primers, 15 cycles) to append sequencing adapters. Libraries were purified with Axygen SPRI beads (target ~400 bp) and sequenced on an Illumina NextSeq 2000 (50 bp paired-end).

### 4.10. Alignments and STARR-Seq Data Analysis

Assay and reporter FASTQ files were aligned to hg38 by Bowtie 2, retaining reads with MAPQ ≥ 30 and excluding centromeric/blacklisted regions. PCR duplicates were marked with Picard. Per-base RPKM coverage tracks were generated using deepTools bamCoverage. CRADLE was applied to correct biases and call regulatory peaks. Differential activity between input (plasmid) and output (RNA) libraries per region was quantified as fold-change by DESeq. Regions exhibiting significant STARR-seq regulatory activity in H295R and COV434 cells were cross-referenced to ATAC-seq data for those lines and ENCODE V4 regulatory annotations.

### 4.11. PCOS Case-Control Variant Association Testing Within STARR-Seq Regions

Genotype data from 983 PCOS cases and 2951 controls (Illumina OmniExpress) were imputed with Minimac4 (TOPMed r2  ≥  0.8) and filtered to variants within active STARR-seq peaks. Logistic regression in PLINK (MAF  >  1%), adjusted for age, BMI, and five ancestry PCs, tested variant associations with PCOS, applying Bonferroni correction based on the number of independent regulatory elements.

### 4.12. Colocalization Testing

Bayesian colocalization between PCOS association statistics (variants with *p*  <  0.3) and GTEx v8 cis-eQTLs across 49 tissues was performed using coloc.abf() with default priors. Posterior probability of a shared causal variant (PP.4  >  0.3) was reported for all tissues, with emphasis on adrenal and ovarian tissues.

Harris, et al. 2023 [10]—Single-Cell RNA-Seq Identifies Pathways and Genes Contributing to the Hyperandrogenemia Associated with Polycystic Ovary Syndrome.

### 4.13. Theca Cell Preparations and Culture

See Harris, et al. 2023 [6] methods above.

### 4.14. Single-Cell RNA Sequencing (scRNA-Seq)

Fourth-passage theca cells from five PCOS and five normal preparations, all unrelated and of European ancestry, were cultured to 85–95% confluence in serum-containing medium under 5% O_2_/5% CO_2_. Cells were rinsed, switched to serum-free defined medium, and incubated for 24 h either untreated or with 20 μM forskolin (Calbiochem, San Diego CA, USA). Post-treatment, cells were harvested by trypsinization in cold DME/F12 with 10% FBS, pelleted at 600× *g*, and resuspended in cryopreservation solution (20% DMSO/50% FBS in DME/F12), (Thermo Fisher Scientific, Waltham, MA, USA). Aliquots (2 mL) were frozen in an isopropanol (SigmaAldich, St. Louis, MO, USA), −80 °C device for 24 h, then stored in liquid nitrogen until shipment to Active Motif (Carlsbad, CA, USA) for cell dilution, RNA isolation, and 10× Chromium single-cell library preparation. Libraries were sequenced on an Illumina NextSeq 500 to generate 91 bp reads.

### 4.15. Cell Ranger Data Processing

Raw scRNA-Seq BCL files were demultiplexed to FASTQ with Cell Ranger v6.1.1’s mkfastq function. The count pipeline was run with default settings to align reads to GRCh37/hg19, filter, and count barcodes and unique molecular identifiers, producing a gene–cell expression matrix.

### 4.16. Seurat Pre-Processing and Quality Control

Cell Ranger output was imported into Seurat [28] v4.1.1 in R v4.1.3. Cells with fewer than 200 detected features or greater than 5% mitochondrial reads were excluded. Data were normalized and the most variable features identified with Seurat’s NormalizeData and FindVariableFeatures functions under default parameters.

### 4.17. STACAS Integration

Semi-supervised integration of individual scRNA-Seq datasets was performed with STACAS [29] v2.0.1 in R, using ovarian-expressed candidate genes (including PCOS loci) as anchor features and known cell labels (affection status and treatment) with high label confidence (0.99). Mitochondrial, ribosomal, heat shock, and cell cycle genes were excluded from highly variable gene selection.

### 4.18. Identification of Differentially Expressed Genes

Seurat’s FindMarkers function (min.pct = 0.01, logfc.threshold = 0.01) conducted Wilcoxon rank-sum tests to identify genes differentially expressed across defined groups, adjusting *p*-values by Bonferroni correction.

## Figures and Tables

**Table 1:** Seurat FindMarkers differential expression analysis of scRNA-seq data from theca cells. Comparisons were performed between each condition and all the other conditions to identify condition specific differential expression patterns. Theca cells were either Normal or PCOS and were either Untreated or Forskolin treated. Wilcoxon rank-sum tests with Bonferroni multiple testing correction were performed to identify differentially expressed genes.

Gene	Condition	Average log2 Fold Change	Adjusted *p* Value	Regulation vs. All Other Conditions
*RAB5B*	Normal Untreated	−0.084	3.51 × 10^−48^	Downregulated
*RAB5B*	Normal Forskolin	0.093	5.70 × 10^−6^	Upregulated
*SUOX*	Normal Forskolin	0.021	2.72 × 10^−16^	Upregulated
*SUOX*	PCOS Forskolin	−0.018	8.28 × 10^−167^	Downregulated
*PA2G4*	Normal Untreated	0.038	0.0047	Upregulated
*ERBB3*	Normal Forskolin	−0.011	1.07 × 10^−252^	Downregulated

## Data Availability

The 10× Cell Ranger filtered feature-barcode matrix data files and the scRNA-seq data are publicly available for download at https://zenodo.org/record/7942968 (accessed on 1 October 2025). These files are the read counts by gene by cell that can be used to analyze single cell data.

## Supplementary Materials

The following supporting information can be downloaded at: https://www.mdpi.com/article/10.3390/ijms262211184/s1.
